# A stabilized MFE reduced-order extrapolation model based on POD for the 2D unsteady conduction-convection problem

**DOI:** 10.1186/s13660-017-1399-7

**Published:** 2017-05-30

**Authors:** Hong Xia, Zhendong Luo

**Affiliations:** 1 School of Control and Computer Engineering, North China Electric Power University, No. 2, Bei Nong Road, Changping District, Beijing, 102206 China; 2 School of Mathematics and Physics, North China Electric Power University, No. 2, Bei Nong Road, Changping District, Beijing, 102206 China

**Keywords:** 65N15, 65N30, stabilized mixed finite element reduced-order extrapolation model, unsteady conduction-convection problem, proper orthogonal decomposition technique, the existence and uniqueness and the stability as well as the convergence

## Abstract

In this study, we devote ourselves to establishing a stabilized mixed finite element (MFE) reduced-order extrapolation (SMFEROE) model holding seldom unknowns for the two-dimensional (2D) unsteady conduction-convection problem via the proper orthogonal decomposition (POD) technique, analyzing the existence and uniqueness and the stability as well as the convergence of the SMFEROE solutions and validating the correctness and dependability of the SMFEROE model by means of numerical simulations.

## Introduction

Let $\Theta\subset \mathbb{R}^{2}$ be an interconnected bounded domain. We are concerned with the following two-dimensional (2D) unsteady conduction-convection problem (see, *e.g.*, [1–3]).

### Problem I

Seek ${\boldsymbol {u}}=(u_{1},u_{y})^{\tau}$, *p*, and *Q* that satisfy

1 $$ \textstyle\begin{cases} {\boldsymbol {u}}_{t}-\mu\Delta{\boldsymbol {u}}+({\boldsymbol {u}}\cdot \nabla){\boldsymbol {u}}+\nabla p= Q{\boldsymbol {j}}, & (x,y,t)\in \Theta\times(0,T), \\ \nabla\cdot{\boldsymbol {u}}=0, & (x,y,t)\in \Theta\times(0,T), \\ Q_{t}- \gamma_{0}^{-1}\Delta Q+ ({\boldsymbol {u}}\cdot \nabla) Q = 0, & (x,y,t)\in \Theta\times(0,T), \\ {\boldsymbol {u}}(x,y,t)= {\boldsymbol {f}}_{0}(x,y,t), \qquad Q(x,y,t)=\varpi (x,y,t), & (x,y,t)\in \partial\Theta\times(0,T), \\ {\boldsymbol {u}}(x,y,0)={\boldsymbol {g}}^{0}(x,y),\qquad Q(x,y,0)=\omega(x,y), & (x,y)\in \Theta, \end{cases} $$

where ${\boldsymbol {u}}=(u_{x},u_{y})^{\tau}$ represents the unknown velocity vector, *p* represents the unknown pressure, *Q* represents the unknown heat energy, *T* is the final moment, ${\boldsymbol {j}}=(0,1)^{\tau}$, $\mu=\sqrt{{\mathit{Pr}/\mathit{Re}}}$, *Pr* is the Prandtl number, *Re* is the Reynolds, $\gamma_{0}=\sqrt{\mathit{Re} \mathit{Pr}}$, and ${\boldsymbol {f}}_{0}(x,y,t)$, ${\boldsymbol {g}}^{0}(x,y)$, $\varpi(x,y,t)$ and $\omega (x,y)$ are four known functions. In order to facilitate theoretical analysis and not to lose universality, we assume that ${\boldsymbol {f}}_{0}(x,y,t)$=${\boldsymbol {g}}^{0}(x,y) =\boldsymbol {0}$ and $\varpi(x,y,t)=0$ in the following study.

Because the 2D unsteady conduction-convection problem is a system of nonlinear PDEs, it usually has no analytic solution so as to have to depend on approximate solutions. Until present, there have been many numerical methods for the 2D unsteady conduction-convection problem (see, *e.g.*, [1–7]), but the stabilized mixed finite element (SMFE) method based on a parameter-free and two local Gauss integrals in [7] is considered as one of the most efficient approaches to solving the 2D unsteady conduction-convection problem. However, the SMFE method includes a lot of unknowns so as to amass a lot of truncated errors and bear very large computational load in the real-world engineering applications. Thus, a key issue is how to decrease the unknowns of the SMFE method so as to ease the truncated error amassing and save the consuming time in the numerical computation but keeping sufficiently high accuracy of numerical solutions.

A number of numerical experiments (see, *e.g.*, [8–21]) have shown that the proper orthogonal decomposition (POD) is a very useful approach to decrease the unknowns for numerical models and ease the truncated error amassing in the numerical computations. But the now available reduced-order numerical methods as stated above were built by means of the POD basis formulated by the classical numerical solutions on all time nodes, before calculating the reduced-order numerical solutions on the same time nodes, which are some vain reduplicated computations. Since 2014, the reduced-order extrapolation MFE models based on POD for the 2D hyperbolic equations, unsteady parabolized Navier-Stokes (NS) equations, and viscoelastic wave equation have been proposed by Luo’s team (see, *e.g.*, [22–24]) to avert the vain reduplicated calculations.

However, as far as we know, there has not been any study where the POD technique is used to establish the SMFE reduced-order extrapolation (SMFEROE) model for the 2D unsteady conduction-convection problem. Therefore, in this article, we devote ourselves to establishing the SMFEROE model via the POD method for the 2D unsteady conduction-convection problem, analyzing the existence and uniqueness and the stability as well as the convergence of the SMFEROE solutions and validating the correctness and dependability of the SMFEROE model by means of numerical simulations.

The major differences between the SMFEROE model and the now available reduced-order extrapolation MFE models based on POD, as stated above, consist in the fact that the conduction-convection problem not only includes the unknown velocity and the unknown pressure, but also has the unknown heat energy coupled nonlinearly with the unknown velocity vector so that it is more complicated than the hyperbolic equations, unsteady parabolized NS equations, and viscoelastic wave equation. Thus, both the modeling of the SMFEROE method and the demonstration of the existence and uniqueness and the stability as well as the convergence of the SMFEROE solutions encounter more difficulties and require more techniques than the now available reduced-order extrapolation MFE models as stated above, but the SMFEROE model has some specific applications. Especially, the SMFEROE model is built by means of the POD basis generated by the SMFE solutions on the initial seldom time nodes, before finding out the SMFEROE solutions at all time nodes by means of the extrapolation iteration so that it does not have reduplicated computation. Consequently, it is development and improvement over the existing models as mentioned above.

The rest of the article is scheduled as follows. In Section 2, we review the SMFE model and the corresponding results for the 2D unsteady conduction-convection problem. In Section 3, we constitute the POD basis by means of the SMFE solutions on the initial seldom time nodes and build the SMFEROE model including seldom unknowns for the 2D unsteady conduction-convection problem by means of the POD basis. Section 4 offers the demonstration of the existence and uniqueness and the stability as well as the convergence of the SMFEROE solutions and the algorithm process for the SMFEROE model. In Section 5, some numerical simulations are supplied to validate the correctness and dependability of the SMFEROE model. Section 6 generalizes the main conclusions.

## Review the fully discrete SMFE model

The following arisen Sobolev spaces as well as their norms are well known (see [25]).

The weak form for the 2D unsteady conduction-convection problem is stated as follows.

### Problem II

Seek $(\boldsymbol {u},p,Q)\in H^{1}(0,T;X)^{2}\times L^{2}(0,T;M)\times H^{1}(0,T;W)$ that satisfies

2 $$ \textstyle\begin{cases} (\boldsymbol {u}_{t},\boldsymbol{\psi})+A(\boldsymbol {u},\boldsymbol{\psi})+A_{1}(\boldsymbol {u},\boldsymbol {u},\boldsymbol{\psi})-B(p,\boldsymbol {\psi})= (Q\boldsymbol {j},\boldsymbol{\psi}), & \forall \boldsymbol{\psi}\in X, \\ B(q,\boldsymbol {u})=0, & \forall q\in M, \\ (Q_{t},\varphi)+D_{0}(Q,\varphi)+ A_{2}(\boldsymbol {u},Q,\varphi) = 0, &\forall\varphi\in W, \\ {\boldsymbol {u}}(x,y,0)=\boldsymbol {0},\qquad Q(x,y,0)=\omega(x,y), & (x,y)\in \Theta, \end{cases} $$

where $X=H^{1}_{0}(\Theta)^{2}$, $M=L_{0}^{2}(\Theta)= \{q\in L^{2}(\Theta ); \int_{\Theta}q\,\mathrm{d}x\,\mathrm{d}y=0 \}$, $W=H^{1}_{0}(\Theta)$, $(\cdot ,\cdot)$ denotes the scalar product of $L^{2}(\Theta)^{2}$ or $L^{2}(\Theta )$, and

$$\begin{gathered} A(\boldsymbol {u},\boldsymbol{\psi})=\mu(\nabla \boldsymbol {u}, \nabla\boldsymbol{\psi}), \quad\forall\boldsymbol {u},\boldsymbol{\psi}\in X;\qquad B(q,\boldsymbol{\psi})=(q, \operatorname{div}\boldsymbol {\psi}), \quad \forall\boldsymbol{\psi}\in X, q\in M, \\ A_{1}(\boldsymbol {u},\boldsymbol{\psi},\boldsymbol{\phi})=0.5 \bigl[ \bigl((\boldsymbol {u}\nabla\boldsymbol{\psi}),\boldsymbol{\phi}\bigr)-\bigl(( \boldsymbol {u}\nabla\boldsymbol{\phi}),\boldsymbol{\psi}\bigr) \bigr], \quad \forall \boldsymbol {u},\boldsymbol{\psi},\boldsymbol{\phi}\in X, \\ A_{2}(\boldsymbol {u},Q,\varphi)=0.5 \bigl[\bigl((\boldsymbol {u}\cdot \nabla Q),\varphi\bigr)-\bigl((\boldsymbol {u}\cdot\nabla\varphi ),Q\bigr) \bigr], \quad \forall\boldsymbol {u}\in X, \forall Q,\varphi\in W, \\ D_{0}(Q,\varphi)= \gamma_{0}^{-1}(\nabla Q,\nabla \varphi ), \quad \forall Q,\varphi \in W. \end{gathered} $$

They have the following properties (see, *e.g.*, [3–7, 26]):

3 $$\begin{aligned}& A_{1}(\boldsymbol {u},\boldsymbol{\psi},\boldsymbol{\phi}) = -A_{1}(\boldsymbol {u},\boldsymbol{\phi},\boldsymbol{\psi});\qquad A_{1}(\boldsymbol {u},\boldsymbol{\psi},\boldsymbol{\psi})=0, \quad \forall \boldsymbol {u},\boldsymbol{\psi}, \boldsymbol{\phi}\in X, \end{aligned}$$

4 $$\begin{aligned}& A_{2}(\boldsymbol {u},Q,\varphi) = -A_{2}( \boldsymbol {u},\varphi,Q);\qquad A_{2}(\boldsymbol {u},\varphi ,\varphi)=0, \quad \forall\boldsymbol {u}\in X, \forall Q,\varphi \in W, \end{aligned}$$

5 $$\begin{aligned}& A(\boldsymbol{\psi},\boldsymbol{\psi})\ge \mu \vert \boldsymbol {\psi} \vert _{1}^{2};\qquad \bigl\vert A(\boldsymbol {u}, \boldsymbol {\psi}) \bigr\vert \le \mu \vert \boldsymbol {u} \vert _{1} \vert \boldsymbol{\psi} \vert _{1}, \quad \forall \boldsymbol {u}, \boldsymbol{\psi}\in X, \end{aligned}$$

6 $$\begin{aligned}& D_{0}(\varphi,\varphi)\ge\gamma_{0}^{-1} \vert \varphi \vert _{1}^{2};\qquad \bigl\vert D_{0}(Q,\varphi) \bigr\vert \le \gamma_{0}^{-1} \vert Q \vert _{1} \vert \varphi \vert _{1}, \quad \forall Q, \varphi\in W, \end{aligned}$$

7 $$\begin{aligned}& \sup_{\boldsymbol{\psi}\in X}\frac{b(q,\boldsymbol{\psi})}{ \vert \boldsymbol{\psi} \vert _{1}}\ge\beta \Vert q \Vert _{0},\quad \forall q\in M, \end{aligned}$$

here *β* is a positive real number. Define

8 $$ N_{0} = \sup_{\boldsymbol {u},\boldsymbol{\psi},\boldsymbol{\phi}\in X}\frac{A_{1}(\boldsymbol {u},\boldsymbol{\psi},\boldsymbol{\phi})}{ \vert \boldsymbol {u} \vert _{1}\cdot \vert \boldsymbol{\psi} \vert _{1}\cdot \vert \boldsymbol{\phi} \vert _{1}}, \qquad \tilde{N}_{0} = \sup_{\boldsymbol {u}\in X,(Q,\varphi)\in W\times W}\frac{A_{2}(\boldsymbol {u}, Q,\varphi)}{ \vert \boldsymbol {u} \vert _{1}\cdot \vert Q \vert _{1}\cdot \vert \varphi \vert _{1}}. $$

The following conclusions about Problem II were proved in [3].

### Theorem 1

*When* $\omega\in L^{2}(\Theta)$ *satisfies* $\Vert \omega \Vert _{0}^{2}\le2\mu^{2}T/(2 N_{0} T^{-1}\exp(T)+\mu\gamma_{0}\tilde {N}_{0}^{2})$, *then Problem* II *has a unique solution that satisfies*

$$\Vert \boldsymbol {u} \Vert _{0}^{2}+\mu \Vert \nabla \boldsymbol {u} \Vert _{L^{2}(L^{2})}^{2}\le T^{2} \Vert \omega \Vert _{0}^{2}\exp(T), \qquad \Vert Q \Vert _{0}^{2}+\gamma_{0}^{-1} \Vert \nabla Q \Vert _{L^{2}(L^{2})}^{2}\le \Vert \omega \Vert _{0}^{2}. $$

For the integer $N>0$, let $k=T/N$ represent the time step, $\Im_{h} = \{K\}$ represent the quasi-uniformity triangle partition of Θ (see [3, 7]), $\mathcal{P}_{1}(K)$ denote the linear polynomial space on *K*, and $(\boldsymbol {u}_{h}^{n}, p_{h}^{n},Q_{h}^{n})$ be the SMFE solutions of $(\boldsymbol {u}(t), p,Q)$ at the time nodes $t_{n}=n k $ ($0\le n\le N$). Then the SMFE model including the parameter-free and two local Gauss integrals can be stated as follows.

### Problem III

Seek $(\boldsymbol {u}_{h}^{n}, p_{h}^{n}, Q_{h}^{n})\in U_{h}\times M_{h}\times W_{h}$ ($n=1, 2, \ldots, N$) that satisfy

9 $$ \textstyle\begin{cases} (\bar{\partial}_{t}\boldsymbol {u}_{h}^{n},\boldsymbol{\psi}_{h}) + A({\boldsymbol {u}}_{h}^{n},\boldsymbol{\psi}_{h}) +A_{1}({\boldsymbol {u}}_{h}^{n},{\boldsymbol {u}}_{h}^{n},\boldsymbol{\psi}_{h})-B(p_{h}^{n},\boldsymbol{\psi}_{h}) = ( Q_{h}^{n}\boldsymbol {j},\boldsymbol{\psi}_{h}), &\forall\boldsymbol {\psi}_{h}\in X_{h}, \\ B(q_{h},\boldsymbol {u}_{h}^{n})+D(p_{h}^{n},q_{h})=0, &\forall q_{h}\in M_{h}, \\ (\bar{\partial}_{t}Q_{h}^{n},\varphi_{h})+D_{0}(Q_{h}^{n},\varphi_{h})+ A_{2}({\boldsymbol {u}}_{h}^{n},Q_{h}^{n},\varphi_{h})=0,& \forall\varphi_{h}\in W_{h}, \\ \boldsymbol {u}_{h}^{0}=\boldsymbol {0}, Q_{h}^{0}=R_{h}\omega (x,y),& (x,y)\in\Theta, \end{cases} $$

where $X_{h}=\{\boldsymbol{\psi}_{h}\in[H_{0}^{1}(\Theta)\cap C(\overline{\Theta })]^{2}; \boldsymbol{\psi}_{h}\vert_{K} \in[\mathcal{ P}_{1}(K)]^{2}, \forall K\in\Im_{h}\}$, $M_{h}= \{\phi_{h}\in M; \phi_{h}\vert_{K}\in \mathcal{P}_{1}(K), \forall K\in\Im_{h} \}$, $W_{h}= \{\varphi_{h}\in H_{0}^{1}(\Theta)\cap C(\overline{\Theta}); \varphi_{h}\vert_{K}\in\mathcal{ P}_{1}(K), \forall K\in\Im_{h} \}$, $\bar{\partial}_{t}\boldsymbol {u}^{n}=(\boldsymbol {u}^{n}-\boldsymbol {u}^{n-1})/k$, $\bar{\partial}_{t}T^{n}=(Q^{n}-Q^{n-1})/k$, $D(p_{h}^{n},q_{h})=\varepsilon\sum_{K\in \Im_{h}}\{\int_{K,2}p_{h}^{n}q_{h}\,\mathrm{d}x\,\mathrm{d}y-\int_{K,1}p_{h}^{n}q_{h}\,\mathrm{d}x\,\mathrm{d}y\} $ ($p_{h},q_{h}\in M_{h}$), *ε* is a positive parameter-free real, $\int_{K,j} g(x,y)\,\mathrm{d}x\,\mathrm{d}y$ ($j = 1, 2$) represent the Gauss integrals on *K* that are exact for *i* degree polynomial $g(x,y) =p_{h}q_{h}$ ($j = 1, 2$), and $R_{h}$ is the Ritz projection from *W* onto $W_{h}$ (see [7]).

Note that, $\forall q_{h}\in M_{h}$, the function $p_{h} \in M_{h}$ should be piecewise constant as $j = 1$. If $\hat{W}_{h}\subset L^{2}(\Theta)$ is the piecewise constant space on $\Im_{h}$ and the operator $\varrho_{h}:L^{2}(\Theta)\rightarrow\hat {W}_{h}$ is defined as follows, $\forall p\in L^{2}(\Theta)$,

10 $$ (p, q_{h}) = (\varrho_{h}p, q_{h}), \quad \forall q_{h}\in\hat{ W}_{h}, $$

then the bilinear functional $D(\cdot,\cdot)$ can be denoted by

11 $$ D(p_{h}, q_{h}) = \varepsilon(p_{h}-\varrho_{h}p_{h}, q_{h}) = \varepsilon(p_{h} -\varrho_{h}p_{h}, q_{h}-\varrho_{h}q_{h}). $$

Furthermore, the operator $\varrho_{h}$ satisfies the following inequalities (see [3, 7, 26]):

12 $$\begin{aligned}& \Vert \varrho_{h}p \Vert _{0}\le C \Vert p \Vert _{0}, \quad \forall p\in L^{2}(\Theta), \end{aligned}$$

13 $$\begin{aligned}& \Vert p-\varrho_{h}p \Vert _{0}\le Ch \Vert p \Vert _{1} , \quad \forall p\in H^{1}(\Theta), \end{aligned}$$

where $C>0$ in this context denotes the constant independent of *h* and *k* that is possibly not the same at different places.

The following conclusions of the existence and uniqueness and the stability as well as the convergence of the SMFE solutions to Problem III have been deduced in [7].

### Theorem 2

*Under the conditions of Theorem* 1, *the SMFE model has only a set of solutions* $\{(\boldsymbol {u}_{h}^{n},p_{h}^{n},Q_{h}^{n}) \}_{n=1}^{N}$ *that satisfies*

14 $$ \bigl\Vert \boldsymbol {u}_{h}^{n} \bigr\Vert _{0}+ \bigl\Vert Q_{h}^{n} \bigr\Vert _{0}+k\sum_{i=1}^{n}\bigl( \bigl\Vert \nabla\boldsymbol {u}_{h}^{i} \bigr\Vert _{0}+ \bigl\Vert \nabla Q_{h}^{i} \bigr\Vert _{0}+ \bigl\Vert p_{h}^{i} \bigr\Vert _{0}\bigr) \le C \Vert \omega \Vert _{0}, $$

*which implies that the set of SMFE solutions* $\{(\boldsymbol {u}_{h}^{n},p_{h}^{n},Q_{h}^{n}) \}_{n=1}^{N}$ *is stable*. *Furthermore*, *if* $\omega\in H^{1}(\Theta)$, $N_{0}\mu^{-1} \Vert \nabla{\boldsymbol {u}}_{h}^{n} \Vert _{0}\le1/4$, *and* $h=O(k)$, *the set of SMFE solutions* $\{(\boldsymbol {u}_{h}^{n},p_{h}^{n},Q_{h}^{n}) \}_{n=1}^{N}$ *satisfies the error estimations*

15 $$\begin{aligned}& k \sum_{i=1}^{n} \bigl[ \bigl\Vert \boldsymbol {u}(t_{i})-\boldsymbol {u}_{h}^{i} \bigr\Vert _{1}+ \bigl\Vert Q(t_{i})-Q_{h}^{i} \bigr\Vert _{1}+ \bigl\Vert p(t_{i})-p_{h}^{i} \bigr\Vert _{0} \bigr] \\& \quad {}+ \bigl\Vert \boldsymbol {u}(t_{n})-\boldsymbol {u}_{h}^{n} \bigr\Vert _{0}+ \bigl\Vert Q(t_{n})-Q_{h}^{n} \bigr\Vert _{0} \leq C\bigl(k+h^{2}\bigr),\quad n=1, 2, \ldots, N, \end{aligned}$$

*where* $(\boldsymbol {u},p, T)$ *represents the generalized solution of Problem* II.

### Remark 1

If only *ω*, *k*, *h*, the Reynolds *Re*, the Prandtl number *Pr*, and the subspaces $X_{h}$, $M_{h}$, and $W_{h}$ are given, a set of SMFE solutions $\{\boldsymbol {u}_{h}^{n},p_{h}^{n},Q_{h}^{n}\}_{n=1}^{n}$ is acquired by Problem III. We choose the initial *L* solutions $(\boldsymbol {u}_{h}^{n},p_{h}^{n}, Q_{h}^{n})$ ($1\le n\le L$, usually, $L\ll N$ and $\sqrt{L}<5$, *e.g.*, $L=20$, $N=4\mbox{,}000$ in the numerical simulations of Section 5) from *N* solutions $(\boldsymbol {u}_{h}^{n},p_{h}^{n}, Q_{h}^{n})$ ($1\le n\le N$) as snapshots.

## Constitute the POD basis and build the SMFEROE model

For the extracted snapshots $(\boldsymbol {u}_{h}^{n},p_{h}^{n},Q_{h}^{n})$ ($1\le n\le L$) in Section 2, set $\boldsymbol {U}_{i}= (\boldsymbol {u}_{h}^{n},p_{h}^{n},Q_{h}^{n})$ ($n=1,2,\ldots,L$) with rank *l* and define the snapshot matrix $\tilde{\boldsymbol {A}}=(\tilde{A}_{ij})_{L\times L}\in R^{ L\times L}$, where ${ \tilde {A}}_{ij}=[(\nabla\boldsymbol {u}_{h}^{i}, \nabla \boldsymbol {u}_{h}^{j})+(p_{h}^{i},p_{h}^{j})+(\nabla Q_{h}^{i}, \nabla Q_{h}^{j})]/L$. Thus, the matrix $\tilde{\boldsymbol {A}}$ is positive semi-definite and has rank *l*, the POD basis $\{{\boldsymbol{\omega}}_{j}\}_{j=1}^{d}$ can be found and has the following results (see, *e.g.*, [10, 12, 14]).

### Lemma 1

*Suppose that the rank of* $\tilde{\boldsymbol {A}}$ *is* *l*, $\lambda_{1} \ge\lambda_{2} \ge\cdots \ge\lambda_{l}>0$ *are the positive eigenvalues of* $\tilde{\boldsymbol {A}}$, *and* $\boldsymbol{\psi}^{1}$, $\boldsymbol {\psi}^{2}$, … , $\boldsymbol{\psi}^{l}$ *are the corresponding orthonormal eigenvectors*. *Then the POD bases are denoted by*

16 $$ {\boldsymbol {\omega}}_{i} =\frac{1}{\sqrt{ L \lambda_{i}}} ( \boldsymbol {U}_{1},\boldsymbol {U}_{2},\ldots,\boldsymbol {U}_{L})\cdot\boldsymbol{\psi}^{i}, \quad 1\leq i\leq d\leq l $$

*and satisfy the following formula*:

17 $$ \frac{1}{ L} \sum_{i=1}^{L} \Biggl\Vert \boldsymbol {U}_{i}- \sum_{j=1}^{d}( \boldsymbol {U}_{i},{\boldsymbol {\omega}}_{j})_{\hat {X}}{ \boldsymbol {\omega}}_{j}\Biggr\Vert ^{2}_{\hat{X}}=\sum _{j=d+1}^{l}\lambda_{j}, $$

*where* $\hat{X}=X\times M\times W$.

Let $\boldsymbol{\omega}_{j}=(\boldsymbol{\omega}_{uj},\omega_{pj}, \omega_{Qj})$ ($j=1,2, \ldots, d$), $X^{d}=\operatorname{span} \{\boldsymbol {\omega}_{u1},\boldsymbol{\omega}_{u2}, \ldots, \boldsymbol{\omega}_{ud} \}$, $M^{d}=\operatorname{span} \{\omega_{p1}, \omega_{p2}, \ldots, \omega _{pd} \}$, and $W^{d}=\operatorname{span} \{\omega_{Q1}, \omega_{Q2}, \ldots, \omega_{Qd} \}$. For $\boldsymbol {u}_{h}\in X_{h}$, $p_{h}\in M_{h}$, and $Q_{h}\in W_{h}$, define, respectively, three projections $P^{d}$: $X_{h}\to X^{d}$, $Z^{d}$: $M_{h}\to M^{d}$, and $R^{d}: W_{h}\to W^{d}$ as follows:

18 $$\begin{aligned}& \bigl(\nabla P^{d}\boldsymbol {u}_{h}, \nabla \boldsymbol {w}_{d}\bigr)= (\nabla\boldsymbol {u}_{h}, \nabla \boldsymbol {w}_{d}),\quad \forall \boldsymbol {w}_{d}\in X^{d}; \end{aligned}$$

19 $$\begin{aligned}& \bigl(Z^{d}p_{h}, p_{d}\bigr)= (p_{h}, q_{d}),\quad \forall q_{d}\in M^{d}; \end{aligned}$$

20 $$\begin{aligned}& \bigl(\nabla R^{d}Q_{h}, \nabla\varpi_{d}\bigr)= (\nabla Q_{h}, \nabla \varpi_{d}),\quad \forall\varpi_{d}\in W^{d}. \end{aligned}$$

Then it is easily known from functional analysis principles (see, *e.g.*, [27]) that there are three extensions $P^{h}$: $X\to X_{h}$, $Z^{h}$: $M\to M_{h}$, and $R^{h}$: $W\to W_{h}$ of $P^{d}$, $Z^{d}$, and $R^{d}$ such that $P^{h}\vert_{X_{h}}=P^{d}: X_{h}\to X^{d}$, $Z^{h}\vert_{M_{h}}=Z^{d}: M_{h}\to M^{d}$, and $R^{h}\vert_{W_{h}}=R^{d}: W_{h}\to W^{d}$ are defined, respectively, by

21 $$\begin{aligned}& \bigl(\nabla P^{h}{\boldsymbol {u}}, \nabla{\boldsymbol {w}}_{h}\bigr)= (\nabla{\boldsymbol {u}}, \nabla{\boldsymbol {w}}_{h}),\quad \forall {\boldsymbol {w}}_{h}\in X_{h}, \end{aligned}$$

22 $$\begin{aligned}& \bigl(Z^{h}p, p_{h} \bigr)= (p, q_{h}),\quad \forall q_{h}\in M_{h}, \end{aligned}$$

23 $$\begin{aligned}& \bigl(\nabla R^{h}Q, \nabla \varpi_{h}\bigr)= (\nabla Q, \nabla \varpi_{h}),\quad \forall \varpi_{h}\in W_{h}, \end{aligned}$$

where $(\boldsymbol {u},p,Q)\in X\times M\times W$. Thanks to (21), (22), and (23), the projections $P^{h}$, $Z^{h}$, and $R^{h}$ all are bounded

24 $$\begin{aligned}& \bigl\Vert \nabla\bigl(P^{h}{\boldsymbol {u}}\bigr) \bigr\Vert _{0}\leq \Vert \nabla{\boldsymbol {u}} \Vert _{0},\quad \forall {\boldsymbol {u}}\in X; \end{aligned}$$

25 $$\begin{aligned}& \bigl\Vert Z^{h}{p} \bigr\Vert _{0}\leq \Vert p \Vert _{0},\quad \forall p\in M; \end{aligned}$$

26 $$\begin{aligned}& \bigl\Vert \nabla\bigl(R^{h}Q \bigr) \bigr\Vert _{0}\leq \Vert \nabla Q \Vert _{0},\quad \forall Q\in W. \end{aligned}$$

Moreover, there are the following results (see [3, 11, 14]):

27 $$\begin{aligned}& \bigl\Vert \boldsymbol {u} -P^{h}\boldsymbol {u} \bigr\Vert _{0}\leq Ch \bigl\Vert \nabla\bigl(\boldsymbol {u} -P^{h}\boldsymbol {u}\bigr) \bigr\Vert _{0}, \quad \forall{ \boldsymbol {u}}\in X; \end{aligned}$$

28 $$\begin{aligned}& \bigl\Vert \boldsymbol {u} -P^{h}\boldsymbol {u} \bigr\Vert _{-1}\leq Ch \bigl\Vert \boldsymbol {u} -P^{h} \boldsymbol {u} \bigr\Vert _{0},\quad \forall{\boldsymbol {u}}\in X; \end{aligned}$$

29 $$\begin{aligned}& \bigl\Vert Q -R^{h}Q \bigr\Vert _{0}\leq Ch \bigl\Vert \nabla\bigl(Q -R^{h}Q\bigr) \bigr\Vert _{0},\quad \forall T\in W; \end{aligned}$$

30 $$\begin{aligned}& \bigl\Vert Q -R^{h}Q \bigr\Vert _{-1} \leq Ch \bigl\Vert Q -R^{h}Q \bigr\Vert _{0},\quad \forall T \in W. \end{aligned}$$

In addition, there are the following conclusions (see, *e.g.*, [3, 12–14]).

### Lemma 2

*The projections* $P^{d}$, $Z^{d}$, *and* $R^{d}$ ($1\leq d\leq l$) *satisfy*, *respectively*,

31 $$\begin{aligned}& \frac{1}{ L} \sum_{n=1}^{L} \bigl[ \bigl\Vert {\boldsymbol {u}}_{h}^{n}-P^{d}{ \boldsymbol {u}}_{h}^{n} \bigl\Vert _{0}^{2}+h^{2} \bigr\Vert \nabla \bigl({\boldsymbol {u}}_{h}^{n}-P^{d}{ \boldsymbol {u}}_{h}^{n}\bigr)\bigr\Vert _{0}^{2} \bigr]\leq Ch^{2}\sum_{j=d+1}^{l} \lambda _{j}; \end{aligned}$$

32 $$\begin{aligned}& \frac{1}{ L} \sum_{n=1}^{L} \bigl\Vert {p}_{h}^{n}-Z^{d}{p}_{h}^{n} \bigr\Vert _{0}^{2}\leq\sum_{j=d+1}^{l} \lambda_{j}; \end{aligned}$$

33 $$\begin{aligned}& \frac{1}{ L} \sum_{n=1}^{L} \bigl[ \bigl\Vert Q_{h}^{n}-R^{d}Q_{h}^{n} \bigr\Vert _{0}^{2}+h^{2} \bigl\Vert \nabla \bigl(Q_{h}^{n}-R^{d}Q_{h}^{n} \bigr) \bigr\Vert _{0}^{2} \bigr]\leq Ch^{2} \sum _{j=d+1}^{l}\lambda_{j}, \end{aligned}$$

*where* $(\boldsymbol {u}_{h}^{n},p_{h}^{n},Q_{h}^{n})\in X_{h}\times M_{h}\times W_{h}$ ($1\le n\le L$) *are the initial* *L* *solutions of Problem* III. *Furthermore*, *the projections* $P^{h}$, $Z^{h}$, *and* $R^{h}$ *hold*, *respectively*, *the following properties*:

34 $$\begin{aligned}& \bigl\Vert \boldsymbol {u}^{n} -P^{h} \boldsymbol {u}^{n} \bigr\Vert _{-1}+h \bigl\Vert \boldsymbol {u}^{n} -P^{h}\boldsymbol {u}^{n} \bigr\Vert _{0}+h^{2} \bigl\Vert \nabla\bigl(\boldsymbol {u}^{n} -P^{h}\boldsymbol {u}^{n}\bigr) \bigr\Vert _{0} \\& \quad \leq Ch^{3},\quad n=1, 2, \ldots, N; \end{aligned}$$

35 $$\begin{aligned}& \bigl\Vert p^{n} -Z^{h}p^{n} \bigr\Vert _{s} \leq Ch^{m-s},\quad n=1, 2, \ldots, N, s=-1,0, m=1,2; \end{aligned}$$

36 $$\begin{aligned}& \bigl\Vert Q^{n} -R^{h}Q^{n} \bigr\Vert _{-1}+h \bigl\Vert Q^{n} -R^{h}Q^{n} \bigr\Vert _{0}+h^{2} \bigl\Vert \nabla \bigl(Q^{n} -R^{h}Q^{n}\bigr) \bigr\Vert _{0} \\& \quad \leq Ch^{3},\quad n=1, 2, \ldots, N, \end{aligned}$$

*where* $(\boldsymbol {u},p,Q)\in H^{2}(\Theta)^{2}\times H^{m}(\Theta)\times H^{2}(\Theta)$ *represents the generalized solution for the* 2*D unsteady conduction*-*convection problem*.

Thus, based on $X^{d}\times M^{d}\times W^{d}$, the SMFEROE formulation for the 2D unsteady conduction-convection problem is set up as follows.

### Problem IV

Find $(\boldsymbol {u}_{d}^{n},p_{d}^{n},Q_{d}^{n})\in X^{d}\times M^{d}\times W^{d}$ ($n=1,2,\ldots,N$) such that

37 $$\begin{aligned}& \bigl(\boldsymbol {u}_{d}^{n},p_{d}^{n},Q_{d}^{n} \bigr) \\& \quad =\sum_{j=1}^{d}\bigl(\bigl(\nabla \boldsymbol {\omega}_{\boldsymbol {u}j}, \nabla\boldsymbol {u}_{h}^{n} \bigr)\boldsymbol{\omega}_{\boldsymbol {u}j}, \bigl(\omega_{pj},p_{h}^{n} \bigr)\omega_{pj}, \bigl(\nabla \omega_{Tj}, \nabla Q_{h}^{n}\bigr)\omega_{Tj}\bigr),\quad n=1, 2, \ldots, L; \end{aligned}$$

38 $$\begin{aligned}& \bigl(\bar{\partial}_{t}\boldsymbol {u}_{d}^{n},\boldsymbol{\psi}_{d}\bigr) +A\bigl({ \boldsymbol {u}}_{d}^{n}, \boldsymbol{\psi}_{d}\bigr) +A_{1}\bigl({\boldsymbol {u}}_{d}^{n},{\boldsymbol {u}}_{d}^{n},\boldsymbol{\psi}_{d}\bigr)-B \bigl(p_{d}^{n},\boldsymbol{\psi}_{d}\bigr) \\& \quad = \bigl( Q_{d}^{n}\boldsymbol {j},\boldsymbol {\psi}_{d}\bigr), \forall\boldsymbol {\psi}_{d}\in X^{d},\quad L+1\le n\le N, \end{aligned}$$

39 $$\begin{aligned}& B\bigl(\boldsymbol {u}_{d}^{n},q_{d} \bigr)+D\bigl(p_{d}^{n},q_{d}\bigr)=0, \quad \forall q_{d}\in M^{d}, L+1\le n\le N, \end{aligned}$$

40 $$\begin{aligned}& \bigl(\bar{\partial}_{t}Q_{d}^{n}, \varpi_{h}\bigr)+D_{0}\bigl(Q_{d}^{n}, \varpi_{d}\bigr)+ A_{2}\bigl({\boldsymbol {u}}_{d}^{n},Q_{d}^{n}, \varpi_{d}\bigr) =0, \quad \forall\varpi_{d}\in W^{d}, L+1\le n\le N, \end{aligned}$$

where $(\boldsymbol {u}_{h}^{n},p_{h}^{n},Q_{h}^{n})\in X_{h}\times M_{h}\times W_{h}$ ($n=1,2, \ldots, L$) are the initial *L* SMFE solutions for Problem III.

### Remark 2

It is easily known that Problem III at each time node contains $4N_{h}$ (here $N_{h}$ represents the number of vertices of triangles in $\Im_{h}$, see [3]) unknowns, but Problem IV at the same time node only has 4*d* ($d\ll l\leq L\ll N\ll N_{h}$) unknowns. For the real-world engineering issues, the number $N_{h}$ of vertices of triangles in $\Im_{h}$ exceeds thousands or even millions; whereas *d* only is the number of the initial seldom eigenvalues and is quite small (for instance, in Section 5, $d=6$, but $N_{h}=3\times136\times10^{4}$). Therefore, Problem IV is the SMFEROE model for the 2D unsteady conduction-convection problem. Especially, Problem IV only uses the initial few known *L* solutions of Problem III to seek other ($N-L$) solutions and does not have reduplicated calculations. In other words, the initial *L* POD-based SMFEROE solutions are gained by means of projecting the initial *L* SMFE solutions into POD basis, while other ($N-L$) SMFEROE solutions are gained by means of extrapolation and iterating equations (38), (39), and (40). Therefore, it is thoroughly different from the now available reduced-order models (see, *e.g.*, [9–14, 20, 28]).

## The existence and uniqueness and the stability as well as the convergence of SMFEROE solutions and the algorithm process for the SMFEROE model

### The existence and uniqueness and the stability as well as the convergence of the SMFEROE solutions

The existence and uniqueness and the stability as well as the convergence of the solutions for the SMFEROE formulation of the 2D unsteady conduction-convection problem have the following main conclusions.

#### Theorem 3

*Under the conditions of Theorem* 2, *Problem* IV *has only a set of solutions* $(\boldsymbol {u}_{d}^{n}, p_{d}^{n},Q_{d}^{n})\in X^{d}\times M^{d}\times W^{d}$ *such that*

41 $$ \bigl\Vert \boldsymbol {u}_{d}^{n} \bigr\Vert _{0}+ \bigl\Vert Q_{d}^{n} \bigr\Vert _{0}+ k\sum_{i=L+1}^{n}\bigl( \bigl\Vert \nabla\boldsymbol {u}_{d}^{i} \bigr\Vert _{0}+ \bigl\Vert p_{d}^{i} \bigr\Vert _{0}+ \bigl\Vert \nabla Q_{d}^{i} \bigr\Vert _{0}\bigr) \le C \Vert \omega \Vert _{0},\quad 1\le n\le N, $$

*which implies that the SMFEROE solutions* $(\boldsymbol {u}_{d}^{n}, p_{d}^{n},Q_{d}^{n})$ ($1\le n\le N$) *of Problem* IV *are stable*. *When* $k=O(h)$ *and* ${N}_{0}\mu^{-1} \Vert \nabla{\boldsymbol {u}}_{d}^{n} \Vert _{0}\le1/4$ ($L+1\le n\le N$), *we have the error estimations*

42 $$\begin{aligned}& \bigl\Vert \boldsymbol {u}_{h}^{n}- \boldsymbol {u}_{d}^{n} \bigr\Vert _{0}+ \bigl\Vert Q_{h}^{n}-Q_{d}^{n} \bigr\Vert _{0}+k \bigl\Vert \nabla \bigl(\boldsymbol {u}_{h}^{n}- \boldsymbol {u}_{d}^{n}\bigr) \bigr\Vert _{0}+k \bigl\Vert \nabla \bigl(Q_{h}^{n}-Q_{d}^{n} \bigr) \bigr\Vert _{0} \\& \quad {}+\sqrt{k} \bigl\Vert p_{h}^{n}-p_{d}^{n} \bigr\Vert _{0}\le CLk \Biggl(\sum_{j=d+1}^{l} \lambda_{j} \Biggr)^{1/2}, \quad 1\le n\le L; \end{aligned}$$

43 $$\begin{aligned}& \bigl\Vert \boldsymbol {u}_{h}^{n}- \boldsymbol {u}_{d}^{n} \bigr\Vert _{0}+ \bigl\Vert Q_{h}^{n}-Q_{d}^{n} \bigr\Vert _{0}+k \bigl\Vert \nabla \bigl(\boldsymbol {u}_{h}^{n}- \boldsymbol {u}_{d}^{n}\bigr) \bigr\Vert _{0}+k \bigl\Vert \nabla \bigl(Q_{h}^{n}-Q_{d}^{n} \bigr) \bigr\Vert _{0} \\& \quad {}+\sqrt{k} \bigl\Vert p_{h}^{n}-p_{d}^{n} \bigr\Vert _{0}\leq C\bigl(k+h^{2}\bigr)+ CLk \Biggl(\sum _{j=d+1}^{l}\lambda_{j} \Biggr)^{1/2},\quad L+1\le n\le N. \end{aligned}$$

#### Proof

When $1\le n\le L$, from (37), we immediately gain unique $(\boldsymbol {u}_{d}^{n},p_{d}^{n}, Q_{d}^{n})\in X^{d}\times M^{d}\times W^{d}$ ($1\le n\le L$). When $L+1\le n\le N$, by using the same approaches as proving Theorem 2 in [7], from (38)-(40) we can gain unique $(\boldsymbol {u}_{d}^{n},p_{d}^{n}, Q_{d}^{n})\in X^{d}\times M^{d}\times W^{d}$ ($L+1\le n\le N$). Thus, Problem IV has only a set of solutions $(\boldsymbol {u}_{d}^{n},p_{d}^{n}, Q_{d}^{n})\in X^{d}\times M^{d}\times W^{d}$ ($1\le n\le N$).

Next, we devote ourselves to proving that (41) holds.

When $1\le n\le L$, by (24)–(26) and Theorem 2, there holds (41).

When $L+1\le n\le N$, by choosing $\boldsymbol{\psi}_{d}=\boldsymbol {u}_{d}^{n}$ in (38) and $q_{d}=p_{d}^{n}$ in (39), noting that there hold $(\rho_{h}p_{d},p_{d})= \Vert \rho_{h}p_{d} \Vert _{0}^{2}$ and $(p_{d}-\rho_{h}p_{d},p_{d}-\rho_{h}p_{d})= \Vert p_{d} \Vert _{0}^{2}- \Vert \rho_{h}p_{d} \Vert _{0}^{2}\ge0$ from (11), and using (3) and Hölder’s and Cauchy’s inequalities, we obtain

44 $$\begin{aligned}& \bigl\Vert \boldsymbol {u}_{d}^{n} \bigr\Vert _{0}^{2}+ k\mu \bigl\Vert \nabla{\boldsymbol {u}}_{d}^{n} \bigr\Vert _{0}^{2}+k \varepsilon\bigl( \bigl\Vert p_{d}^{n} \bigr\Vert _{0}^{2}- \bigl\Vert \varrho_{h}p_{h}^{d} \bigr\Vert _{0}^{2}\bigr) \\& \quad =\bigl(\boldsymbol {u}_{d}^{n-1},\boldsymbol {u}_{d}^{n}\bigr)+ \bigl( Q_{d}^{n}\boldsymbol {j},\boldsymbol {u}_{d}^{n}\bigr) \\& \quad \leq \frac{1}{2}\bigl( \bigl\Vert \boldsymbol {u}_{d}^{n} \bigr\Vert _{0}^{2}+ \bigl\Vert \boldsymbol {u}_{d}^{n-1} \bigr\Vert _{0}^{2}\bigr)+ C{k} \bigl\Vert Q_{d}^{n} \bigr\Vert _{-1}^{2}+ \frac{k\mu}{2} \bigl\Vert \nabla {\boldsymbol {u}}_{d}^{n} \bigr\Vert _{0}^{2}. \end{aligned}$$

It follows from (44) that

45 $$ \bigl\Vert \boldsymbol {u}_{d}^{n} \bigr\Vert _{0}^{2}- \bigl\Vert \boldsymbol {u}_{d}^{n-1} \bigr\Vert _{0}^{2}+ 2k\mu \bigl\Vert \nabla {\boldsymbol {u}}_{d}^{n} \bigr\Vert _{0}^{2}+2k\varepsilon\bigl( \bigl\Vert p_{d}^{n} \bigr\Vert _{0}^{2}- \bigl\Vert \varrho_{h}p_{h}^{d} \bigr\Vert _{0}^{2}\bigr)\leq Ck \bigl\Vert Q_{d}^{n} \bigr\Vert _{-1}^{2}. $$

If $p_{d}^{n}\neq0$, then it is easily known from (11) that $\Vert p_{d}^{n} \Vert _{0}^{2}> \Vert \varrho_{h}p_{d}^{n} \Vert _{0}^{2}$. Thus, there is a positive real number $\delta\in(0,1)$ that satisfies $\delta \Vert p_{d}^{n} \Vert _{0}^{2}\ge \Vert \varrho_{h}p_{d}^{n} \Vert _{0}^{2}$. By summing (45) from $L+1$ to *n* simplified, we have

46 $$ \bigl\Vert \boldsymbol {u}_{d}^{n} \bigr\Vert _{0}^{2}+ k\sum_{i=L+1}^{n} \bigl( \bigl\Vert \nabla\boldsymbol {u}_{d}^{i} \bigr\Vert _{0}^{2}+ \bigl\Vert p_{d}^{i} \bigr\Vert _{0}^{2}\bigr) \le C \bigl\Vert \boldsymbol {u}_{d}^{L} \bigr\Vert _{0}^{2}+ Ck\sum _{i=L+1}^{n} \bigl\Vert Q_{d}^{i} \bigr\Vert _{-1}^{2}. $$

Taking a square root for (46) and utilizing $(\sum_{i=1}^{n}a_{i}^{2} )^{1/2}\ge\sum_{i=1}^{n} \vert a_{i} \vert /\sqrt{n}$ yield

47 $$ \bigl\Vert \boldsymbol {u}_{d}^{n} \bigr\Vert _{0}+ k\sum_{i=L+1}^{n} \bigl( \bigl\Vert \nabla\boldsymbol {u}_{d}^{i} \bigr\Vert _{0}+ \bigl\Vert p_{d}^{i} \bigr\Vert _{0}\bigr) \le C \Biggl( \bigl\Vert \boldsymbol {u}_{d}^{L} \bigr\Vert _{0}^{2}+ k\sum_{i=L+1}^{n} \bigl\Vert Q_{d}^{i} \bigr\Vert _{-1}^{2} \Biggr)^{1/2}. $$

By choosing $\varphi_{d}=Q_{d}^{n}$ in (40) and by making use of (4) and Hölder ’s and Cauchy’s inequalities, we obtain

48 $$ \bigl\Vert Q_{d}^{n} \bigr\Vert _{0}^{2}+ \frac{2k}{\gamma_{0}} \bigl\Vert \nabla Q_{d}^{n} \bigr\Vert _{0}^{2}\le \bigl\Vert Q_{d}^{n-1} \bigr\Vert _{0}^{2}. $$

Summing (48) from L+1 to *n* yields

49 $$ \bigl\Vert Q_{d}^{n} \bigr\Vert _{0}^{2}+ \frac{2k}{\gamma_{0}}\sum _{i=L+1}^{n} \bigl\Vert \nabla Q_{d}^{i} \bigr\Vert _{0}^{2}\le \bigl\Vert Q^{L} \bigr\Vert _{0}^{2}. $$

By extracting a square root for (49), making use of $(\sum_{i=1}^{n}a_{i}^{2} )^{1/2}\ge\sum_{i=1}^{n} \vert a_{i} \vert /\sqrt{n}$ and (41) when $n=L$, and then simplifying, we obtain

50 $$ \bigl\Vert Q_{d}^{n} \bigr\Vert _{0}+ k\sum_{i=L+1}^{n} \bigl\Vert \nabla Q_{d}^{i} \bigr\Vert _{0} \le C \Vert \omega \Vert _{0}. $$

By noting that $\Vert \cdot \Vert _{-1}\le C \Vert \cdot \Vert _{0}$ and by using (41) when $n=L$, from (47) and (49), we obtain

51 $$ \bigl\Vert \boldsymbol {u}_{d}^{n} \bigr\Vert _{0}+ k\sum_{i=L+1}^{n} \bigl( \bigl\Vert \nabla\boldsymbol {u}_{d}^{i} \bigr\Vert _{0}+ \bigl\Vert p_{d}^{i} \bigr\Vert _{0}\bigr) \le C \Vert \omega \Vert _{0}. $$

Combining (50) with (51) yields that (41) holds when $L+1\le n\le N$. If $p_{d}^{n}=0$, (41) is distinctly correct.

When $1\le n\le L$, with Lemma 2 and (37), we immediately obtain (42).

When $L+1\le n\le N$, by subtracting Problem IV from Problem III choosing $\boldsymbol{\psi}_{h}=\boldsymbol{\psi}_{d}$, $q_{h}=q_{d}$, and $\varphi_{h}=\varphi_{d}$, we acquire

52 $$\begin{aligned}& \bigl(\boldsymbol {u}_{h}^{n}- \boldsymbol {u}_{d}^{n}, \boldsymbol{\psi}_{d}\bigr)+ kA \bigl({\boldsymbol {u}}_{h}^{n}- {\boldsymbol {u}}_{d}^{n}, \boldsymbol{\psi}_{d} \bigr)+kA_{1}\bigl( {\boldsymbol {u}}_{h}^{n}, { \boldsymbol {u}}_{h}^{n},\boldsymbol {\psi}_{d}\bigr) -kA_{1}\bigl( {\boldsymbol {u}}_{d}^{n}, { \boldsymbol {u}}_{d}^{n}, \boldsymbol{\psi}_{d}\bigr) \\& \quad {}-kB\bigl(p_{h}^{n}-p_{d}^{n},\boldsymbol {\psi}_{d}\bigr)= k\bigl(\bigl( Q_{h}^{n}- Q_{d}^{n}\bigr)\boldsymbol {j}, \boldsymbol{\psi}_{d} \bigr) +\bigl(\boldsymbol {u}_{h}^{n-1}-\boldsymbol {u}_{d}^{n-1}, \boldsymbol{\psi}_{d}\bigr),\quad \forall \boldsymbol{\psi}_{d}\in X^{d}, \end{aligned}$$

53 $$\begin{aligned}& b\bigl(q_{d},\boldsymbol {u}_{h}^{n}- \boldsymbol {u}_{d}^{n}\bigr)+\varepsilon \bigl(p_{h}^{n}-p_{d}^{n}- \varrho_{h}\bigl(p_{h}^{n}-p_{d}^{n} \bigr), q_{d}-\varrho_{h}q_{d}\bigr) =0,\quad \forall q_{d}\in M^{d}, \end{aligned}$$

54 $$\begin{aligned}& \bigl(Q_{h}^{n}-Q_{d}^{n}, \varphi_{d}\bigr)+kD_{0}\bigl(Q_{h}^{n}-Q_{d}^{n}, \varphi_{d}\bigr)+kA_{2}\bigl({\boldsymbol {u}}_{h}^{n}, Q_{h}^{n}, \varphi_{d}\bigr)-kA_{2}\bigl( {\boldsymbol {u}}_{d}^{n}, Q_{d}^{n}, \varphi_{d}\bigr) \\& \quad =\bigl(Q_{h}^{n-1}-Q_{d}^{n-1}, \varphi_{d}\bigr),\quad\forall\varphi_{d}\in W^{d}, L+1\le n\le N. \end{aligned}$$

Let $\boldsymbol {e}^{n}=P^{d}\boldsymbol {u}_{h}^{n}-\boldsymbol {u}_{d}^{n}$, $\boldsymbol {f}^{n}=\boldsymbol {u}_{h}^{n}-P^{d}\boldsymbol {u}_{h}^{n}$, $\eta ^{n}=Z^{d}p_{h}^{n}-p_{d}^{n}$, and $\xi^{n}=p_{h}^{n}-Z^{d}p_{h}^{n}$. First, from (18), (52), and (53), we obtain

55 $$ \begin{aligned}[b] &\bigl\Vert \boldsymbol {e}^{n} \bigr\Vert _{0}^{2}+k \mu \bigl\Vert \nabla\boldsymbol {e}^{n} \bigr\Vert _{0}^{2}\\ &\quad =\bigl(P^{d} \boldsymbol {u}_{h}^{n}-\boldsymbol {u}_{d}^{n}, \boldsymbol {e}^{n}\bigr)+kA\bigl(P^{d}\boldsymbol {u}_{h}^{n}-\boldsymbol {u}_{d}^{n}, \boldsymbol {e}^{n}\bigr) \\ &\quad =-\bigl(\boldsymbol {f}^{n},\boldsymbol {e}^{n}\bigr)+\bigl( \boldsymbol {u}_{h}^{n}-\boldsymbol {u}_{d}^{n}, \boldsymbol {e}^{n}\bigr)+kA\bigl(\boldsymbol {u}_{h}^{n}- \boldsymbol {u}_{d}^{n},\boldsymbol {e}^{n}\bigr) \\ &\quad =\bigl(\boldsymbol {f}^{n-1}-\boldsymbol {f}^{n},\boldsymbol {e}^{n}\bigr)+kB\bigl(p_{h}^{n}-p_{d}^{n}, \boldsymbol {e}^{n}\bigr)-kA_{1}\bigl( {\boldsymbol {u}}_{h}^{n}, {\boldsymbol {u}}_{h}^{n}, \boldsymbol {e}^{n}\bigr) \\ &\qquad {}+kA_{1}\bigl( {\boldsymbol {u}}_{d}^{n},{ \boldsymbol {u}}_{d}^{n}, \boldsymbol {e}^{n}\bigr)+ \bigl(\boldsymbol {e}^{n-1}, \boldsymbol {e}^{n}\bigr) +k\bigl( \bigl( Q_{h}^{n}- Q_{d}^{n}\bigr) \boldsymbol {j}, \boldsymbol {e}^{n}\bigr) \\ &\quad =\bigl(\boldsymbol {f}^{n-1}-\boldsymbol {f}^{n},\boldsymbol {e}^{n}\bigr)-kA_{1}\bigl({\boldsymbol {u}}_{h}^{n}, {\boldsymbol {u}}_{h}^{n}, \boldsymbol {e}^{n} \bigr)+kA_{1}\bigl({\boldsymbol {u}}_{d}^{n},{ \boldsymbol {u}}_{d}^{n}, \boldsymbol {e}^{n}\bigr) \\ &\qquad {}+k\bigl(\bigl( Q_{h}^{n}- Q_{d}^{n} \bigr)\boldsymbol {j}, \boldsymbol {e}^{n}\bigr) +\bigl(\boldsymbol {e}^{n-1},\boldsymbol {e}^{n}\bigr)+k B\bigl(\xi^{n}, \boldsymbol {e}^{n}\bigr)+kB\bigl(\eta,\boldsymbol {e}^{n}\bigr) \\ &\qquad {}-2k\varepsilon\bigl(p_{h}^{n}-p_{d}^{n}- \varrho _{h}\bigl(p_{h}^{n}-p_{d}^{n} \bigr), \eta^{n}-\varrho_{h}\eta\bigr) \\ &\quad \le C\bigl(k^{-1} \bigl\Vert \boldsymbol {f}^{n-1}- \boldsymbol {f}^{n} \bigr\Vert _{-1}^{2}\bigr)+4k \mu^{-1} \bigl\Vert \eta^{n} \bigr\Vert _{0}^{2}+Ck \bigl\Vert \xi^{n} \bigr\Vert _{0}^{2} \\ &\qquad {}+\frac{k\mu}{8} \bigl\Vert \nabla\boldsymbol {e}^{n} \bigr\Vert _{0}^{2}+\frac{1}{2} \bigl\Vert \boldsymbol {e}^{n-1} \bigr\Vert _{0}^{2}+\frac{1}{2} \bigl\Vert \boldsymbol {e}^{n} \bigr\Vert _{0}^{2}-2k \varepsilon\bigl( \bigl\Vert \eta^{n} \bigr\Vert _{0}^{2}- \Vert \varrho_{h}\eta \Vert _{0}^{2}\bigr) \\ &\qquad {}-kA_{1}\bigl( {\boldsymbol {u}}_{h}^{n}, { \boldsymbol {u}}_{h}^{n}, \boldsymbol {e}^{n}\bigr) +kA_{1}\bigl( {\boldsymbol {u}}_{d}^{n}, { \boldsymbol {u}}_{d}^{n}, \boldsymbol {e}^{n}\bigr) +k \bigl(\bigl( Q_{h}^{n}- Q_{d}^{n}\bigr) \boldsymbol {j}, \boldsymbol {e}^{n}\bigr). \end{aligned} $$

Next, when $N_{0}\mu^{-1} \Vert \nabla\boldsymbol {\boldsymbol {u}}_{h}^{n} \Vert _{0}\le1/4$ and $N_{0}\mu^{-1} \Vert \nabla {\boldsymbol {u}}_{d}^{n} \Vert _{0}\le1/4$ ($L+1\le n\le N$), with the properties of $A_{1}(\cdot,\cdot,\cdot)$, Hölder’s and Cauchy’s inequalities, and Lemma 2, we gain

56 $$ kA_{1}\bigl( {\boldsymbol {u}}_{d}^{n}, {\boldsymbol {u}}_{d}^{n}, \boldsymbol {e}^{n} \bigr)-kA_{1}\bigl( {\boldsymbol {u}}_{h}^{n}, { \boldsymbol {u}}_{h}^{n}, \boldsymbol {e}^{n}\bigr) \le Ck \bigl\Vert \nabla\boldsymbol {f}^{n} \bigr\Vert _{0}^{2}+\frac{k\mu }{4} \bigl\Vert \nabla\boldsymbol {e}^{n} \bigr\Vert _{0}^{2}. $$

And then, with Hölder’s and Cauchy’s inequalities, we gain

57 $$ k\bigl(\bigl( Q_{h}^{n}- Q_{d}^{n}\bigr)\boldsymbol {j}, \boldsymbol {e}^{n} \bigr)\le Ck \bigl\Vert Q_{h}^{n}- Q_{d}^{n} \bigr\Vert _{-1}^{2}+ \frac{k\mu}{8} \bigl\Vert \nabla\boldsymbol {e}^{n} \bigr\Vert _{0}^{2}. $$

If $\eta^{n}\neq0$, it is accessible to get $\Vert \eta^{n} \Vert _{0}^{2}> \Vert \varrho_{h}\eta \Vert _{0}^{2}$ from (11). Thus, there exists a positive real number $\delta\in(0,1)$ that satisfies $\delta \Vert \eta^{n} \Vert _{0}^{2}\ge \Vert \varrho_{h}\eta \Vert _{0}^{2}$. When $k=O(h)$, by choosing $\varepsilon=5\mu^{-1}(1-\delta)^{-1}$, combining (55) with (56) and (57), using (28), and then simplifying, we acquire

58 $$ \begin{gathered}[b] \bigl\Vert \boldsymbol {e}^{n} \bigr\Vert _{0}^{2}- \bigl\Vert \boldsymbol {e}^{n-1} \bigr\Vert _{0}^{2}+k \bigl\Vert \nabla \boldsymbol {e}^{n} \bigr\Vert _{0}^{2}+k \bigl\Vert \eta^{n} \bigr\Vert _{0}^{2} \\ \quad \le Ck\bigl( \bigl\Vert \nabla\boldsymbol {f}^{n} \bigr\Vert _{0}^{2}+ \bigl\Vert \boldsymbol {f}^{n-1} \bigr\Vert _{0}^{2}+ \bigl\Vert \xi^{n} \bigr\Vert _{0}^{2}\bigr) + Ck \bigl\Vert Q_{h}^{n}- Q_{d}^{n} \bigr\Vert _{-1}^{2}. \end{gathered} $$

Summing (58) from $L+1$ to *n* yields

59 $$\begin{aligned}& \bigl\Vert \boldsymbol {e}^{n} \bigr\Vert _{0}^{2}+k\sum_{i=L+1}^{n} \bigl( \bigl\Vert \nabla\boldsymbol {e}^{i} \bigr\Vert _{0}^{2}+ \bigl\Vert \eta^{n} \bigr\Vert _{0}^{2}\bigr) \\& \quad \le C \bigl\Vert \boldsymbol {e}^{L} \bigr\Vert _{0}^{2}+Ck \sum_{i=L}^{n} \bigl( \bigl\Vert \nabla\boldsymbol {f}^{i} \bigr\Vert _{0}^{2}+ \bigl\Vert \xi^{i} \bigr\Vert _{0}^{2}+ \bigl\Vert Q_{h}^{i}- Q_{d}^{i} \bigr\Vert _{-1}^{2}\bigr). \end{aligned}$$

By extraction of a square root to (59) and making use of $(\sum_{i=1}^{n}a_{i}^{2} )^{1/2}\ge\sum_{n=1}^{n} \vert a_{i} \vert /\sqrt{n}$, we gain

60 $$\begin{aligned}& \bigl\Vert \boldsymbol {e}^{n} \bigr\Vert _{0} +k \sum_{i=L+1}^{n}\bigl( \bigl\Vert \nabla\boldsymbol {e}^{i} \bigr\Vert _{0}+ \bigl\Vert \eta^{i} \bigr\Vert _{0}\bigr) \\& \quad \le C \Biggl[ \bigl\Vert \boldsymbol {e}^{L} \bigr\Vert _{0}^{2}+ k\sum_{i=L}^{n} \bigl( \bigl\Vert \nabla\boldsymbol {f}^{i} \bigr\Vert _{0}^{2}+ \bigl\Vert \xi^{i} \bigr\Vert _{0}^{2}+ \bigl\Vert Q_{h}^{i}- Q_{d}^{i} \bigr\Vert _{-1}^{2}\bigr) \Biggr]^{1/2}. \end{aligned}$$

Moreover, from Lemma 2 as well as Theorem 2, we acquire

61 $$\begin{aligned}& \begin{aligned}[b] k\sum_{i=L}^{n} \bigl\Vert \nabla\boldsymbol {f}^{i} \bigr\Vert _{0} &\le k \sum_{i=L}^{n}\bigl[ \bigl\Vert \nabla \bigl(\boldsymbol {u}_{h}^{i}-\boldsymbol {u}(t_{i}) \bigr) \bigr\Vert _{0}+ \bigl\Vert \nabla \bigl(\boldsymbol {u}(t_{i})-\boldsymbol {u}^{i}\bigr) \bigr\Vert _{0} \\ &\quad {}+ \bigl\Vert \nabla\bigl(\boldsymbol {u}^{i}-P^{h} \boldsymbol {u}^{i}\bigr) \bigr\Vert _{0}+ \bigl\Vert \nabla( P^{h}\bigl(\boldsymbol {u}^{i}-\boldsymbol {u}_{h}^{i}\bigr) \bigr\Vert _{0}\bigr] \\ &\le C\bigl(h^{2}+k\bigr), \end{aligned} \end{aligned}$$

62 $$\begin{aligned}& \begin{aligned}[b]k\sum_{i=L}^{n} \bigl\Vert \xi^{i} \bigr\Vert _{0} &\le k\sum _{i=L}^{n} \bigl[ \bigl\Vert p_{h}^{i}-p(t_{i}) \bigr\Vert _{0}+ \bigl\Vert p(t_{i})-p^{i} \bigr\Vert _{0}\\ &\quad {}+ \bigl\Vert p^{i}-Z^{h}p^{i} \bigr\Vert _{0}+ \bigl\Vert Z^{h}\bigl(p^{i}-p_{h}^{i} \bigr) \bigr\Vert _{0}\bigr] \\ &\le C\bigl(h^{2}+k\bigr). \end{aligned} \end{aligned}$$

Combining (61) and (62) with (60) and using Lemma 2 and (42) when $n=L$ yield

63 $$\begin{aligned}& \bigl\Vert \boldsymbol {e}^{n} \bigr\Vert _{0} +k \sum _{i=L+1}^{n}\bigl( \bigl\Vert \nabla \boldsymbol {e}^{i} \bigr\Vert _{0}+ \bigl\Vert \eta^{i} \bigr\Vert _{0}\bigr) \\& \quad \le C\bigl(k+h^{2}\bigr)+CLk \Biggl(\sum_{j=d+1}^{l} \lambda_{j} \Biggr)^{1/2}+C \Biggl[ k\sum _{i=L}^{n} \bigl\Vert Q_{h}^{i}- Q_{d}^{i} \bigr\Vert _{-1}^{2} \Biggr]^{1/2}. \end{aligned}$$

Let $F_{n}=Q_{h}^{n}-Z^{d}Q_{h}^{n}$, $E_{n}=Z^{d}Q_{h}^{n}-Q_{d}^{n}$. First, by making use of (54) and Lemma 2, we acquire

64 $$ \begin{gathered}[b] \Vert E_{n} \Vert _{0}^{2}+k \gamma_{0}^{-1} \Vert \nabla {E}_{n} \Vert _{0}^{2} \\ \quad =(E_{n}, {E}_{n})+kD_{0}( {E}_{n}, {E}_{n}) \\ \quad=-(F_{n}, {E}_{n})+kD_{0}\bigl(Z^{d} Q_{h}^{n}- Q_{h}^{n}, {E}_{n} \bigr) +\bigl[\bigl(Q_{h}^{n}-Q_{d}^{n}, {E}_{n}\bigr)+kD_{0}\bigl( Q_{h}^{n}- Q_{d}^{n}, {E}_{n}\bigr)\bigr] \\ \quad=-(F_{n}, {E}_{n})+kA_{2}\bigl( {\boldsymbol {u}}_{d}^{n}, Q_{d}^{n}, {E}_{n} \bigr)-kA_{2}\bigl({\boldsymbol {u}}_{h}^{n}, Q_{h}^{n}, {E}_{n}\bigr) + \bigl(Q_{h}^{n-1}-Q_{d}^{n-1}, {E}_{n}\bigr) \\ \quad=(F_{n-1}-F_{n}, {E}_{n})+kA_{2}\bigl( {\boldsymbol {u}}_{d}^{n}, Q_{d}^{n}, {E}_{n}\bigr)-kA_{2}\bigl( {\boldsymbol {u}}_{h}^{n}, Q_{h}^{n}, {E}_{n}\bigr) +(E_{n-1}, {E}_{n}) \\ \quad\le Ck^{-1}\bigl( \Vert F_{n} \Vert _{-1}^{2}+ \Vert F_{n-1} \Vert _{-1}^{2}\bigr)+Ck \Vert F_{n} \Vert _{0}^{2})+\frac{k}{4\gamma_{0}} \Vert \nabla {E}_{n} \Vert _{0}^{2} \\ \qquad{}+kA_{2}\bigl( {\boldsymbol {u}}_{d}^{n}, Q_{d}^{n}, {E}_{n}\bigr)-kA_{2}\bigl( { \boldsymbol {u}}_{h}^{n}, Q_{h}^{n}, {E}_{n}\bigr)+ \frac{1}{2} \Vert E_{n-1} \Vert _{0}^{2}+\frac{1}{2} \Vert E_{n} \Vert _{0}^{2}. \end{gathered} $$

And then, when $N_{0}\mu^{-1} \Vert \nabla{\boldsymbol {u}}_{d}^{n} \Vert _{0}\le1/4$ ($n=1, 2, \ldots, N$), with Lemma 2, (4), and Hölder’s and Cauchy’s inequalities, we have

65 $$ kA_{2}\bigl( {\boldsymbol {u}}_{d}^{n}, Q_{d}^{n}, {E}_{n} \bigr)-kA_{2}\bigl( {\boldsymbol {u}}_{h}^{n}, Q_{h}^{n}, {E}_{n}\bigr)\le\frac{k}{4\gamma_{0}} \Vert \nabla {E}_{n} \Vert _{0}^{2}+Ck \Vert \nabla F_{n} \Vert _{0}^{2}. $$

Combining (64) with (65) and using Lemma 2, Theorems 1 and 2, the same technique as (61) yield that

66 $$ \Vert E_{n} \Vert _{0}^{2}+k \gamma_{0}^{-1} \Vert \nabla {E}_{n} \Vert _{0}^{2} \le Ck\bigl(h^{4}+k^{2}\bigr)+ \Vert E_{n-1} \Vert _{0}^{2}. $$

Summing (66) from $L+1$ to *n* yields that

67 $$ \Vert E_{n} \Vert _{0}^{2}+k \gamma_{0}^{-1}\sum_{i=L+1}^{n} \Vert \nabla {E}_{i} \Vert _{0}^{2} \le Cnk \bigl(h^{4}+k^{2}\bigr)+ C \Vert E_{L} \Vert _{0}^{2}. $$

By extraction of a square root to (67) and making use of $(\sum_{i=1}^{n}a_{i}^{2} )^{1/2}\ge\sum_{i=1}^{n} \vert a_{i} \vert /\sqrt{n}$ and (42), we acquire

68 $$ \Vert E_{n} \Vert _{0}+k\sum _{i=L+1}^{n} \Vert \nabla {E}_{i} \Vert _{0} \le C\bigl(h^{2}+k\bigr)+CLk \Biggl(\sum _{j=d+1}^{l}\lambda_{j} \Biggr)^{1/2}. $$

With the triangle inequality of norm, (68), and Lemma 2, we acquire

69 $$ \bigl\Vert Q_{h}^{n}-Q_{d}^{n} \bigr\Vert _{0}+k\sum_{i=L+1}^{n} \bigl\Vert \nabla\bigl(Q_{h}^{i}-Q_{d}^{i} \bigr) \bigr\Vert _{0}\le C\bigl(h^{2}+k\bigr)+CLk \Biggl( \sum_{j=d+1}^{l}\lambda_{j} \Biggr)^{1/2}. $$

By combining (63) with (69) and making use of Lemma 2, we acquire

70 $$ \begin{gathered}[b] \bigl\Vert \boldsymbol {u}_{h}^{n}-\boldsymbol {u}_{d}^{n} \bigr\Vert _{0}+k\sum_{i=L+1}^{n}\bigl( \bigl\Vert \nabla\bigl(\boldsymbol {u}_{h}^{i}-\boldsymbol {u}_{d}^{i}\bigr) \bigr\Vert _{0}+ \bigl\Vert p_{h}^{i}-p_{d}^{i} \bigr\Vert _{0}\bigr) \\ \quad \le C\bigl(k+h^{2}\bigr) +CLk \Biggl(\sum _{j=d+1}^{l}\lambda_{j} \Biggr)^{1/2}. \end{gathered} $$

Combining (69) with (70) yields (43). When $\eta^{n}=0$, (43) is distinctly correct. Thus, the argument of Theorem 3 is accomplished. □

By combining Theorem 2 with Theorem 3, we immediately acquire the following conclusion.

#### Theorem 4

*Under the conditions of Theorems* 2 *and* 3, *the SMFEROE solutions* $(\boldsymbol {u}_{d}^{n}, p_{d}^{n}, Q_{d}^{n})$ *for Problem* IV *hold the error estimations*

$$\begin{gathered} k\sum_{i=1}^{n} \bigl[ \bigl\Vert \nabla\bigl(\boldsymbol {u}(t_{i})-\boldsymbol {u}_{d}^{i}\bigr) \bigr\Vert _{0}+ \bigl\Vert \nabla \bigl(Q(t_{i})-Q_{d}^{i}\bigr) \bigr\Vert _{0}\bigr]+ \bigl\Vert p(t_{i})-p_{d}^{i} \bigr\Vert _{0}] \\ \quad {}+ \bigl\Vert \boldsymbol {u}(t_{n})-\boldsymbol {u}_{d}^{n} \bigr\Vert _{0}+ \bigl\Vert Q(t_{n})-Q_{d}^{n} \bigr\Vert _{0}\le C\bigl(k+h^{2}\bigr)+ CLk \Biggl(\sum _{j=d+1}^{l}\lambda_{j} \Biggr)^{1/2}, \end{gathered} $$

*where* $(\boldsymbol {u},p,T)$ *represents the generalized solution for the* 2*D unsteady conduction*-*convection problem*.

#### Remark 3

The factor $Lk (\sum_{j=d+1}^{l}\lambda _{j} )^{1/2}$ in Theorems 3 and 4 is caused by reduced-order for Problem II, it can be used as a suggestion choosing the amount of POD basis, that is, we only need to choose *d* that satisfies $k^{2}L^{2}\sum_{j=d+1}^{l}\lambda_{j} = O(k^{2},h^{4})$, we can acquire the SMFROE solutions satisfying the accuracy requirement.

### The algorithm process for the SMFEROE model

The algorithm process for the SMFEROE model can be carried out according to the next seven steps.

Step 1: Extract the snapshots $\boldsymbol {U}_{n}(x, y)=(\boldsymbol {u}_{h}^{n},p_{h}^{n},Q_{h}^{i})$ ($1\le n\le L$ and $L\ll N$) from the initial *L* SMFE solutions.
Step 2: Compile the snapshot matrix $\tilde {\boldsymbol {A}}=(\tilde{{A}}_{ij})_{L\times L}$, where $\tilde{{A}}_{ij}=[(\nabla\boldsymbol {u}_{h}^{i},\nabla \boldsymbol {u}_{h}^{j}) +(p_{h}^{i}, p_{h}^{j}) +(\nabla Q_{h}^{i},\nabla Q_{h}^{j})]/L$.
Step 3: Find the positive eigenvalues $\lambda_{1}\ge \lambda_{2}\ge\cdots\ge\lambda_{l}>0$ ($l=\dim\{\boldsymbol {U}_{1}, \boldsymbol {U}_{2}, \ldots, \boldsymbol {U}_{L}\}$) of $\tilde {\boldsymbol {A}}$ and the corresponding eigenvectors $\boldsymbol {v}^{j}=(a_{1}^{j},a_{2}^{j},\ldots,a_{L}^{j})^{\tau}$ ($j=1,2,\ldots,l$).
Step 4: For *h*, *k*, and error *ν* needed, determine the amount *d* of POD basis that satisfies $k^{2}+h^{4}+L^{2}k^{2}\sum_{j=d+1}^{l}\lambda_{j}\le\nu^{2}$.
Step 5: Constitute the POD basis $\boldsymbol{\omega}_{j}(x,y)=(\boldsymbol{\omega}_{uj}(x,y),\omega _{pj}(x,y),\omega_{Qj}(x,y)) =\sum_{j=1}^{L}a_{i}^{j} (\boldsymbol {u}_{h}^{i}, p_{h}^{i}, Q_{h}^{i} )/\sqrt{L\lambda_{j}}$ ($1\le j\le d$).
Step 6: Let $X^{d}= \operatorname{span}\{\boldsymbol{\omega}_{u1}(x, y), \boldsymbol{\omega}_{u2}(x,y), \ldots, \boldsymbol {\omega}_{u d}(x,y)\}$, $M^{d}=\operatorname{span} \{\omega_{p1}(x, y), \omega _{p2}(x,y), \ldots, \omega_{pd}(x,y)\}$, and $W^{d}=\operatorname{span}\{\omega_{Q1}(x, y), \omega_{Q2}(x,y), \ldots, \omega_{Qd}(x,y)\}$. Solving Problem IV gives the SMFEROE solutions $(\boldsymbol {u}_{d}^{n},p_{d}^{n}, Q_{d}^{n})$ ($1\le n\le N$).
Step 7: If $\Vert\boldsymbol {u}_{d}^{n-1}-\boldsymbol {u}_{d}^{n} \Vert_{0}\ge\Vert\boldsymbol {u}_{d}^{n}-\boldsymbol {u}_{d}^{n+1} \Vert_{0}$, $\Vert p_{d}^{n-1} -p_{d}^{n} \Vert_{0}\ge\Vert p_{d}^{n}-p_{d}^{n+1} \Vert_{0}$, and $\Vert Q_{d}^{n-1}-Q_{d}^{n} \Vert_{0}\ge\Vert Q_{d}^{n}-Q_{d}^{n+1} \Vert_{0}$ ($L\le n\le N-1$), then $(\boldsymbol {u}_{d}^{n},p_{d}^{n}, Q_{d}^{n})$ ($1\le n\le N$) are the SMFEROE solutions satisfying the accuracy requirement. Else, namely, if $\Vert\boldsymbol {u}_{d}^{n-1}-\boldsymbol {u}_{d}^{n} \Vert_{0}<\Vert\boldsymbol {u}_{d}^{n}-\boldsymbol {u}_{d}^{n+1}\Vert_{0}$ or $\Vert p_{d}^{n-1}-p_{d}^{n} \Vert_{0}< \Vert p_{d}^{n}-p_{d}^{n+1} \Vert_{0}$ or $\Vert Q_{d}^{n-1}-Q_{d}^{n} \Vert_{0}< \Vert Q_{d}^{n}-Q_{d}^{n+1} \Vert_{0} $ ($n=L, L+1,\ldots, N-1$), put $\boldsymbol {U}_{n+j-L}=(\boldsymbol {u}_{d}^{j},p_{d}^{j}, Q_{d}^{j})$ ($j=0, 1, \ldots, L-1$), return to Step 2.

## Numerical simulations

In the following, we use the numerical simulations to validate the correctness and dependability of the SMFEROE model for the 2D unsteady conduction-convection problem.

**Figure 1:**
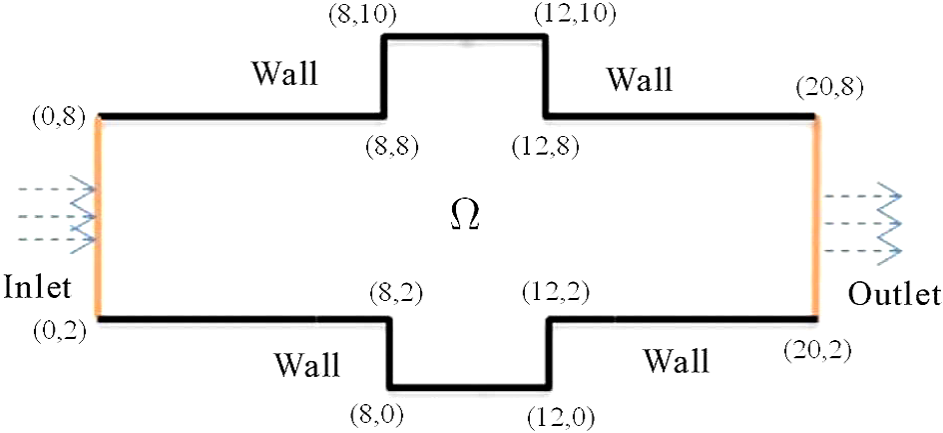
**The computational domain and the initial boundary values.**

The computational domain Θ̄ is composed of the channel of width 6 and length 20 holding two same rectangular cavities of width 2 and length 4 at the top and bottom of the channel (see Figure 1). We first partition Θ̄ into several quadrates whose side length equals $\triangle x =\triangle y= 0.01$. Then we partition each quadrate into two triangles by linking diagonal in the same orientation and form the triangularizations $\Im_{h}$ with $h=\sqrt{2}\times10^{-2}$. Choose $\varepsilon=1$, $\mathit{Pr}=7$, and $\mathit{Re}=1\mbox{,}000$. Besides the inflow velocity $\boldsymbol {u}=(0.1(y-2)(8-y),0)^{T}$ ($x=0$ and $2\le y\le8$) on the left boundary, the other initial and boundary values are chosen as 0. We choose $k=0.01$ in order to satisfy the condition $k=O(h)$.

**Figure 2:**
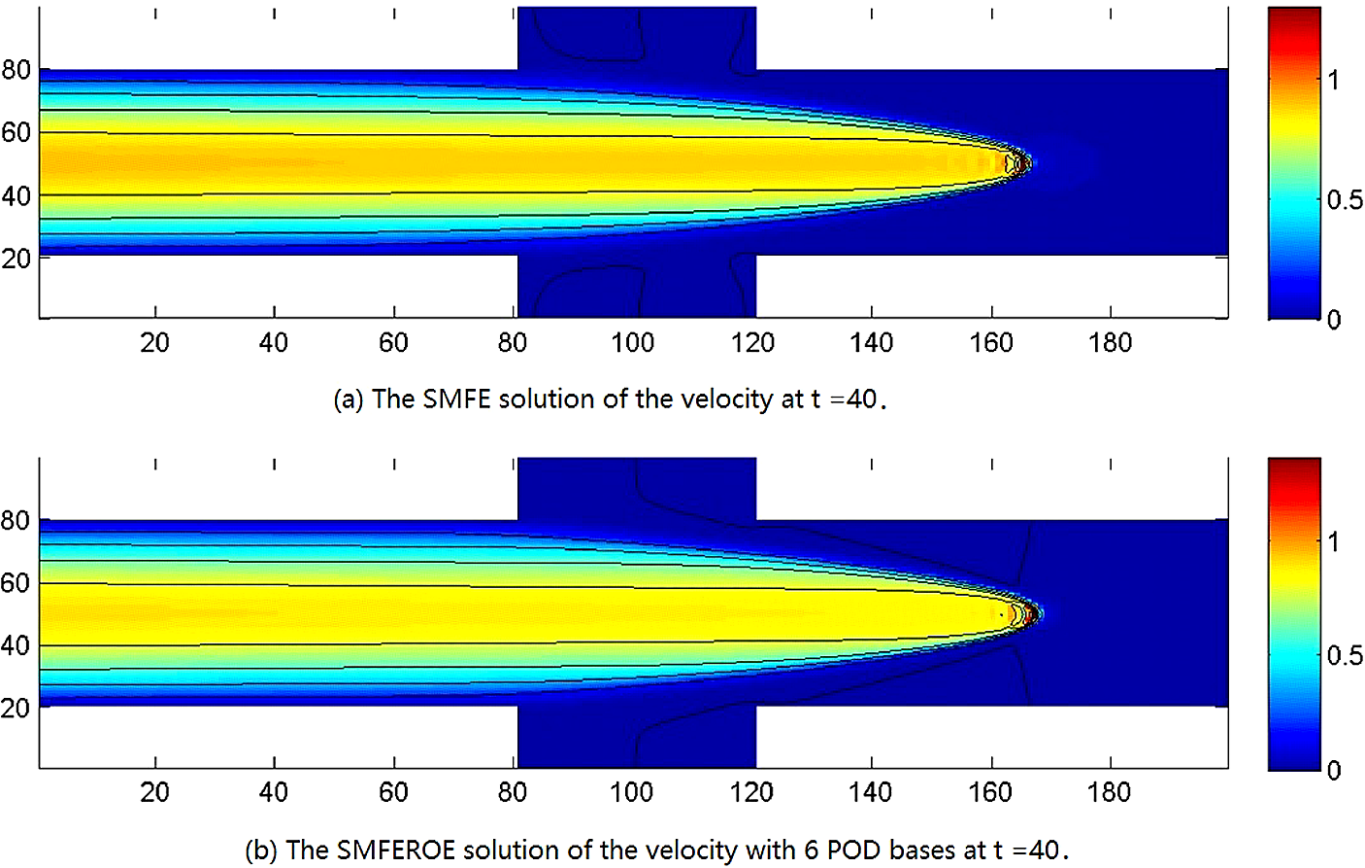
**The numerical solutions of the velocity.** **(a)** The SMFE solution of the velocity ***u*** at $t =40$ when $\mathit{Re}=1\mbox{,}000$ and $\mathit{Pr}=7$. **(b)** The SMFEROE solution of the velocity ***u*** with 6 POD bases at time $t =40$ when $\mathit{Re}=1\mbox{,}000$ and $\mathit{Pr}=7$.

**Figure 3:**
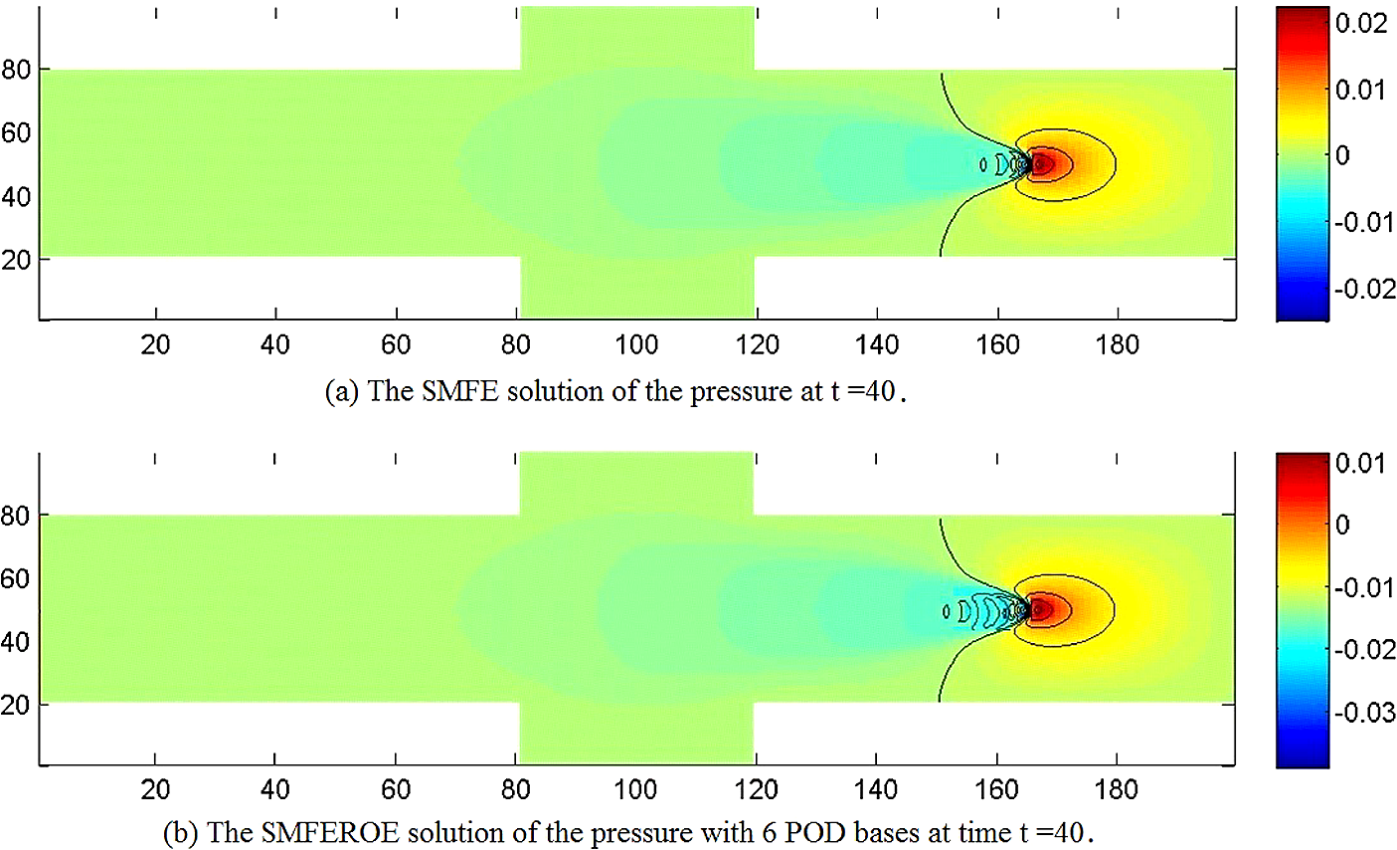
**The numerical solutions of the pressure.** **(a)** The SMFE solution of the pressure *p* at $t =40$ when $\mathit{Re}=1\mbox{,}000$ and $\mathit{Pr}=7$. **(b)** The SMFEROE solution of the pressure *p* with 6 POD bases at time $t =40$ when $\mathit{Re}=1\mbox{,}000$ and $\mathit{Pr}=7$.

**Figure 4:**
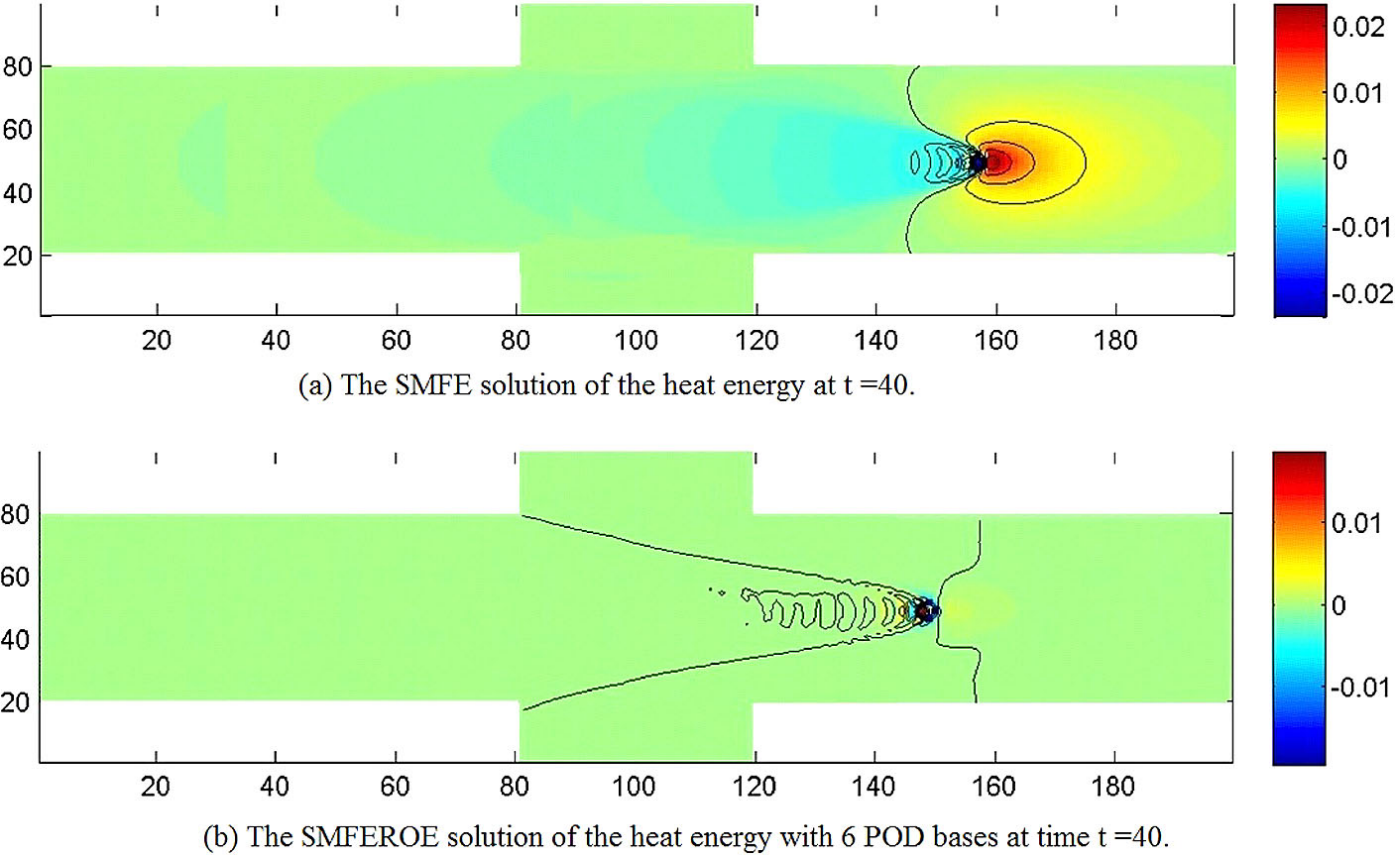
**The numerical solutions of the heat energy.** **(a)** The SMFE solution of the heat energy *Q* at $t =40$ when $\mathit{Re}=1\mbox{,}000$ and $\mathit{Pr}=7$. **(b)** The SMFEROE solution of the heat energy *Q* with 6 POD bases at time $t =40$ when $\mathit{Re}=1\mbox{,}000$ and $\mathit{Pr}=7$.

We first extracted 20 SMFE solutions $(\boldsymbol {u}_{h}^{n},p_{h}^{n},Q_{h}^{n})$ ($n=1, 2, \ldots, 20$) from the SMFE model to constitute snapshots $\boldsymbol {U}_{n}=(\boldsymbol {u}_{h}^{n},p_{h}^{n},Q_{h}^{n})$ ($1\le n\le20$). Next, we sought out 20 eigenvectors and 20 eigenvalues arrayed in a non-increasing order according to Step 3 in Section 4.2. It was achieved by calculation that $Lk (\sum_{j=7}^{20}\lambda_{j} )^{1/2}\leq3\times10^{-2}$ when $k=0.01$ and $L=20$, which implies that it is only necessary to choose the initial 6 eigenvectors $(\boldsymbol{\omega}_{uj}, \omega_{pj},\omega_{Qj})$ ($1\le j\le6$) to generate subspaces $X^{d}\times M^{d}\times W^{d}$. And then, we found the SMFEROE solutions $(\boldsymbol {u}_{d}^{n},p_{d}^{n},Q_{d}^{n})$ ($n=4\mbox{,}000$, *i.e.*, at $t=40$) by means of the SMFEROE model according to seven steps in Section 4.2, which are drawn in (b) graphs of Figures 2-4, but the corresponding SMFE solutions of the velocity, pressure, and heat energy obtained from the SMFE model are drawn in (a) graphs of Figures 2-4 at $t=40$, *i.e.*, $n=4\mbox{,}000$, respectively. Every pair of graphs in Figures 2-4 are basically identical, respectively, but because the SMFEROE model eases the truncated error amassing in the calculating procedure, the SMFEROE solutions acquired from the SMFEROE model are better than the SMFE solutions from the SMFE model. Especially, the numerical results of the pressure and heat energy of the SMFEROE solutions are far better than those of the SMFE solutions.

**Figure 5:**
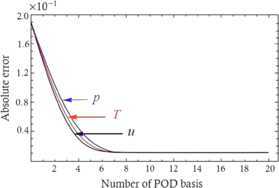
**Absolute error for** $\pmb{\mathit{Re} =1\mbox{,}000}$ **and** $\pmb{\mathit{Pr}=7}$ **when POD basis is different and at the time level** $\pmb{t =40}$ **.**

Figure 5 exhibits the errors between the SMFEROE solutions acquired from the SMFEROE model adopting the different amount of the POD basis and the SMFE solutions gained from the SMFE model when $t=40$, *i.e.*, $n=4\mbox{,}000$, $\mathit{Pr}=7$, and $\mathit{Re}=1\mbox{,}000$. It is shown that the numerical computational conclusions are accorded with the theoretical cases since the numerical and theoretical errors both do not exceed $4\times10^{-2}$.

Further, by comparing the SMFE model with the SMFEROE model with 6 POD bases implementing the numerical simulations when $t=40$, $\mathit{Pr}=7$, and $\mathit{Re}=1\mbox{,}000$, we find that the SMFE model includes $4\times136\times 10^{4}$ unknowns on every time node and the elapsed time is about 180 minutes, but the SMFEROE model with 6 POD bases only has $4\times6$ unknowns at the same time node and the corresponding elapsed time is no more than 60 seconds, *i.e.*, the elapsed time of the SMFE model is 180 times more than that of the SMFEROE model with 6 POD bases. Thus, the SMFEROE model can immensely decrease the elapsed time and ease the computational load so that it could immensely ease the truncated error amassing in the calculation procedure. This implies that the SMFEROE model is effective and dependable for solving the 2D unsteady conduction-convection problem.

## Conclusions

In this article, we have established the SMFEROE model for the 2D unsteady conduction-convection problem by means of the POD technique. We first extract the initial seldom *L* ($L\ll N$) SMFE solutions for the 2D unsteady conduction-convection problem and formulate the snapshots. Next, we constitute the POD basis by the snapshots by means of the POD technique. And then, the subspaces generated with the initial seldom POD basis substitute the MFE subspaces in the SMFE model in order to establish the SMFEROE model for the 2D unsteady conduction-convection problem. Finally, we analyze the existence and uniqueness and the stability as well as the convergence of the SMFEROE solutions for the 2D unsteady conduction-convection problem and supply the algorithm process for the SMFEROE model. Comparing the numerical simulation results of the SMFEROE solutions with the SMFE solutions validates the dependability and correctness of the SMFEROE model.
